# Environmental Change-Dependent Inherited Epigenetic Response

**DOI:** 10.3390/genes10010004

**Published:** 2018-12-21

**Authors:** Alexandra Weyrich, Dorina Lenz, Jörns Fickel

**Affiliations:** 1 Evolutionary Genetics (Dept.2), Leibniz Institute for Zoo and Wildlife Research (IZW), Alfred-Kowalke-Str. 17, D-10315 Berlin, Germany; dlenz@izw-berlin.de (D.L.); fickel@izw-berlin.de (J.F.); 2 Institute for Biochemistry and Biology, Potsdam University, Karl-Liebknecht-Str. 24-25, 14476 Potsdam, Germany

**Keywords:** DNA methylation, exposure, wild mammal species, inheritance, plasticity, adaptation, RRBS

## Abstract

Epigenetic modifications are a mechanism conveying environmental information to subsequent generations via parental germ lines. Research on epigenetic responses to environmental changes in wild mammals has been widely neglected, as well as studies that compare responses to changes in different environmental factors. Here, we focused on the transmission of DNA methylation changes to naive male offspring after paternal exposure to either diet (~40% less protein) or temperature increase (10 °C increased temperature). Because both experiments focused on the liver as the main metabolic and thermoregulation organ, we were able to decipher if epigenetic changes differed in response to different environmental changes. Reduced representation bisulfite sequencing (RRBS) revealed differentially methylated regions (DMRs) in annotated genomic regions in sons sired before (control) and after the fathers’ treatments. We detected both a highly specific epigenetic response dependent on the environmental factor that had changed that was reflected in genes involved in specific metabolic pathways, and a more general response to changes in outer stimuli reflected by epigenetic modifications in a small subset of genes shared between both responses. Our results indicated that fathers prepared their offspring for specific environmental changes by paternally inherited epigenetic modifications, suggesting a strong paternal contribution to adaptive processes.

## 1. Introduction

The same genotype can give rise to different phenotypes under different environmental conditions [1,2]. However, we still understand little of the underlying mechanisms providing the genetic plasticity for this phenotypic diversity. Promising candidates are epigenetic modifications. They increase genetic plasticity [3] as their patterns are modified in response to changing environmental conditions, thereby impacting the regulation of gene expression [4,5]. DNA methylation is an epigenetic modification that can be transmitted with the DNA to subsequent generations and may prepare offspring for environments experienced by their parents [6,7,8,9]. Promoter methylation has often been strongly associated with gene repression [10,11], while intragenic methylation may have either a gene-silencing or gene-activating function [12,13]. So far, epigenetic alterations in response to changing environmental conditions have mainly been studied in highly inbred lab strains that were kept under artificial environments for many generations [7,8,9,14]. The results therefore lack the functional understanding of responses to ecological cues (e.g., climate change) in naturally occurring, genetically heterogeneous species [15,16].

Although studies in wild animals could provide such understanding, those studies are rare [15]. This is especially critical as current environmental changes (e.g., climate change) force wild animals to cope more rapidly with their consequences (e.g., changing temperatures and rainfall). In terms of changing weather patterns, they are shifting the composition of floral communities, followed by changes in the composition of faunal communities. The few epigenetic studies on wild species exposed to ecologically relevant impacts such as changes in ambient temperature or food quality have been carried out in plants [17], honey bees [18], fish [19], wild guinea pigs [20], and baboons [21]. In each of these studies, an impact on methylation patterns was detected after the studied environmental factor had changed. For example, in baboons, researchers detected differences in genome-wide methylation patterns (mainly in promoter and enhancer regions close to metabolism-related genes) between lodge-feeding and wild-feeding baboons [21]. Another ecological impact has been seen in vertebrates, where the sex of the offspring is determined by a temperature-dependent process, such as in turtles and crocodiles. In the European sea bass fish, an increase in temperature leads to masculinization of females and an increased methylation at the promoter of the gonadal aromatase *cyp19a* gene, which is the enzyme converts androgen to oestrogen [22]. With increasing global temperatures, offspring may thus become all-female or, as in other species, all-male [23]. If epigenetic mechanisms also have an evolutionary relevance by affecting fitness is still a matter of debate [24,25].

**Figure 1:**
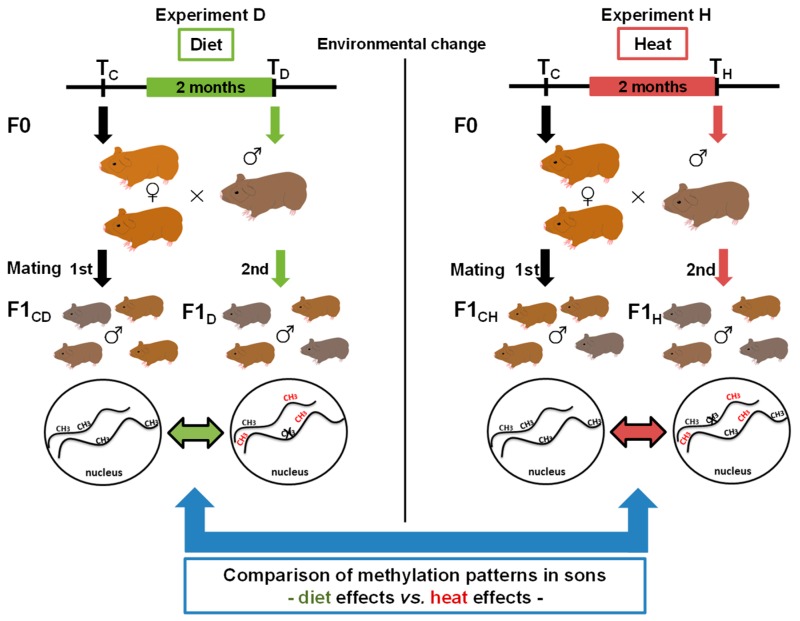
Experimental setup and study aim. Male wild guinea pigs (*n* = 5) were exposed for two months either to an altered diet (“experiment D”, left side, green) or to an increase in temperature (“experiment H”, right side, red). Each male mated with the same two females before (first mating, T_C_) and after the period of exposure (second mating, T_D_ and T_H_). Sons sired before the father’s environmental change represented the control groups (F1_CD_ = control diet and F1_CH_ = control heat), and sons sired afterwards represented the diet (F1_D_) or heat (F1_H_) group. We then analyzed DNA methylation patterns before and after the treatment to identify epigenetic inheritance. In the current study, we aimed to compare genes and gene pathways of the two environmental factors by comparing epigenetic diet effects versus heat effects.

Whether the epigenetic response of an animal depends on the environmental factor the animal was exposed to, or if the response is rather general and largely independent from the type of environmental change, has not been studied yet, either in model species or in wild mammals. Previously, we hypothesized a treatment-specific epigenetic response [26], which we aimed to investigate within the current study using the wild guinea pig *Cavia aperea* as a model species. Wild guinea pigs are distributed nearly South American-wide, and they can survive and reproduce in high and low altitudes with changing vegetation and temperature: They are thus regarded to be a generalist species [27,28]. In previous studies on wild guinea pigs, we showed that paternal epigenetic effects in terms of DNA methylation changes occurred in naïve wild guinea pig sons sired by fathers that had been exposed to changes in two different environmental factors (one factor change per experiment), a low protein diet (diet (D), “experiment D”) and an increase in ambient temperature (heat (H), “experiment H”) (Figure 1).

So far, changes in methylation patterns in sons after paternal heat treatments have been described as a combination of results from different organs and in pairwise comparison with the father generation. To decipher inherited responses to changes in different environmental factors, we here focused on the methylation changes in the livers of sons whose fathers had either experienced changes in ambient temperature or diet.

We focused on the liver because both environmental factors induce a physiological response in the liver. The liver is the main metabolic organ, acting on changes in nutrition as well as in regaining homeostasis when the temperature changes. This allows a direct comparison of the epigenetic responses to two environmental factors within the (same) functionally responding organ. This is additionally relevant to studies using the liver as a target organ to measure epigenetic or transcriptomics responses because the total response might erroneously be interpreted as a factor-specific response, where in fact only parts of the response might be factor-specific.

Both independent experiments were performed in parallel (at the same facility and in the same time period), each with a group of five adult male guinea pigs of the same age, which were kept under stable conditions except for the 2-month exposure to a specific environmental change (“experiment D”, “experiment H”).

This unique experimental setup (two experiments distinguished only by the environmental factor that was changed in them) allowed us to test the three following hypotheses (with the null hypothesis being “no changes in methylation pattern”):

**Hypothesis 1** **(H1).** Environmental changes cause a general epigenetic response independent of the environmental factor that has changed. Under this hypothesis, changes in methylation patterns should be similar in both experiments and should comprise the same genes and gene pathways.

**Hypothesis 2** **(H2).** Changes in different environmental factors cause different, factor-specific epigenetic responses. Under this hypothesis, changes in methylation patterns should be different in both experiments and should involve different genes and gene pathways.

**Hypothesis 3** **(H3).** A combination of H1 and H2: Changes in different environmental factors cause both a factor-independent general and a factor-specific response. Under this hypothesis, changes in methylation patterns should involve both shared and different genes and gene pathways.

To test these hypotheses, we used the results of “experiment D” and of “experiment H” and searched both for changed methylation patterns specific to each experiment (and thus indicative of a factor-specific response) and for changed methylation patterns that involved the same genes and gene pathways (indicative of a general response).

## 2. Materials and Methods

### 2.1. Animal Housing and Experimental Setup

Both experiments were carried out in parallel at the Leibniz Institute for Zoo and Wildlife Research (IZW) field station in Niederfinow, Germany, and have been previously described in detail [26,29]. All husbandry and experimental procedures were approved of by the German Committee of Animal Welfare in Research (permit no. V3-2347-35-2011). For each experiment, five F0 males (but no females) were exposed to either a change in diet (“experiment D”, see below) or to an elevated ambient temperature (“experiment H”, see below) for 62 days. The length of this period reflects a full cycle of spermatogenesis in guinea pigs [30,31]. In both of the experiments, each male was mated twice with two females, once before and once after the change in the environmental factor, and livers (Ls) of F1 sons (F1Ls) were harvested at 7 days of age. We obtained wild guinea pigs (*C. aperea*) from Prof. Fritz Trillmich and Dr. Anja Gunther from the University of Bielefeld, where the animals were kept in cages, and set up housing accordingly at the IZW field station in Niederfinow, Germany. After arrival at the field station, the animals were allowed to “settle in” for a 2-month period to exclude potential transport and captivity effects on epigenetic patterns. Male guinea pig control groups (F0_CD_ and F0_CH_) were kept at the same temperature and were fed the same diet. Both groups had the same mating setup (one male mated with the same two females in mating 1 and 2). To keep any potential “aging effect” as small as possible, we narrowed the time window between the two matings to the shortest period possible, which, in order to avoid reproductive stress for the females, was 6 months.

In “experiment D”, we aimed to assess the paternal effects of a low-protein diet on male offspring. Male wild guinea pigs (F0 fathers, *n* = 5, labeled A–E) were fed a low-protein diet in which pellets had 42% less protein content than in the standard maintenance diet. DNA from the livers of the sons sired before (control group; F1L_CD_, *n* = 15) and after the diet change (diet group; F1L_D_, *n* = 17) were pooled by father and were then analyzed for their methylation patterns via reduced representation bisulfite sequencing (RRBS) [32] (Link S1).

In “experiment H”, we aimed to assess the paternal effect of an increase in temperature on male offspring. Adult male wild guinea pigs (F0 fathers, *n* = 5, labeled F–J) were kept on heating plates at 30 °C (10 °C higher than ambient temperature). Using heating plates allowed us to apply the temperature increase just to male guinea pigs of the treatment group. DNA from the livers of the sons sired before (control; F1L_CH_, *n* = 16) and after the heat treatment (F1L_H_, *n* = 18) were analyzed for their methylation patterns via RRBS, and methylation results were grouped by father for further analysis [26,29].

### 2.2. Assessment of Differentially Methylated Regions and Comparison of Gene Methylation

Differentially methylated regions (DMRs) were identified and assessed separately per experiment (for details, see Reference [20]). The assessment was performed by pairwise comparisons of “control” sons (F1L_CD_ and F1L_CH_) with the respective groups of sons after treatment (either F1L_D_ or F1L_H_), where sons were grouped according to their fathers (“experiment D”: A–E; “experiment H”: F–J). To assess potential false positives, DMRs were validated by random shuffle tests. We shuffled the methylation ratios of F1L_CH_ versus F1L_H_ (“experiment H”) and the methylation ratios of F1L_CD_ versus F1L_D_ (“experiment D”), respectively, 100 times per father, which resulted in <2 DMRs per calculation by chance. DMRs that overlapped with gene promoters or coding sequences (CDS) were separated into three groups: (1) Genes whose methylation was only impacted after a change in diet (“experiment D”), (2) genes that were differentially methylated after a temperature increase only (“experiment H”), and (3) genes whose methylation patterns changed in both experiments (by comparing gene lists resulting from (1) and (2)).

### 2.3 Display of Interactions of Differentially Methylated Genes

We then submitted all genes that were part of at least one DMR to the web-based STRING database (https://string-db.org/, Version 10.5, [33,34]), which combines known and predicted protein–protein association data such as direct (physical) interactions, as well as indirect (functional) interactions, as long as both are specific and biologically meaningful [33]. To assess the probability (considering the available evidence) that an interaction between proteins does exist, STRING calculates a stringency score (which lies between zero and one). For that, STRING uses available experimental data on protein–protein interaction information and imports known pathways and protein complexes from curated databases [34]. Additional interaction predictions are derived from four sources: (1) Systematic co-expression analysis, (2) detection of shared selective signals across genomes, (3) automated text mining of the scientific literature, and (4) computational transfer of interaction knowledge between organisms based on gene orthology. Changing the stringency score changes the number of interactions identified and displayed (higher score = fewer interactions, lower score = more interactions), accompanied by reassessments of the significance *p*-values. Here, we assumed that differentially methylated genes also differentially regulate gene expression, affecting the downstream protein expression. Therefore, we applied STRING to identify physiological pathways that might be regulated by exposure to changing environmental factors via DNA methylation.

## 3. Results

### 3.1. Total Changes in DNA Methylation in Sons

**Figure 2:**
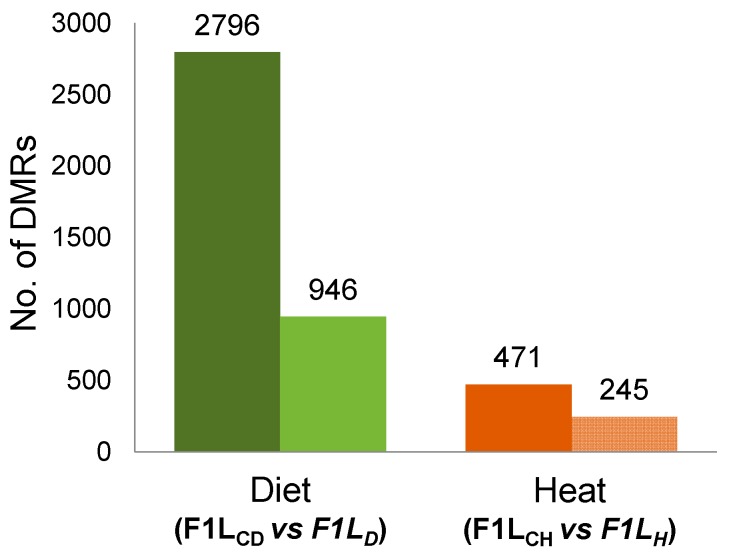
Total number and number of annotated differentially methylated regions (DMRs) after “experiment D” and after “experiment H”; total number of DMRs (dark-colored bars) and the number of annotated DMRs (light-colored bars) between control sons (F1L_C_) and sons sired by fathers either fed a low-protein diet (F1L_D_, left two bars in green) or exposed to a prolonged temperature increase (F1L_H_, right two bars in red). We considered DMRs to be “annotated” when they overlapped with gene coding sequences (CDS), promoters, or CpG islands (CGIs).

To test hypotheses H1–H3, we compared the RRBS data to search for regions with DNA methylation changes (DMRs) in the livers (Ls) of F1 sons (F1L) from both experiments (“experiment D”, F1L_CD_ vs F1L_D_, and “experiment H”, F1L_CH_ vs F1L_H_). In both experiments, we detected DMRs (Figure 2) between the father-sorted groups of sons that had been sired (by the same fathers and mothers) before and after the environmental factors were experimentally changed for fathers.

We focused on DMRs within genes, including both promoter and CDS, which were observed in response to changes in either one of the environmental factors.

### 3.2. Environmental Factor-Specific Epigenetic Response

**Figure 3:**
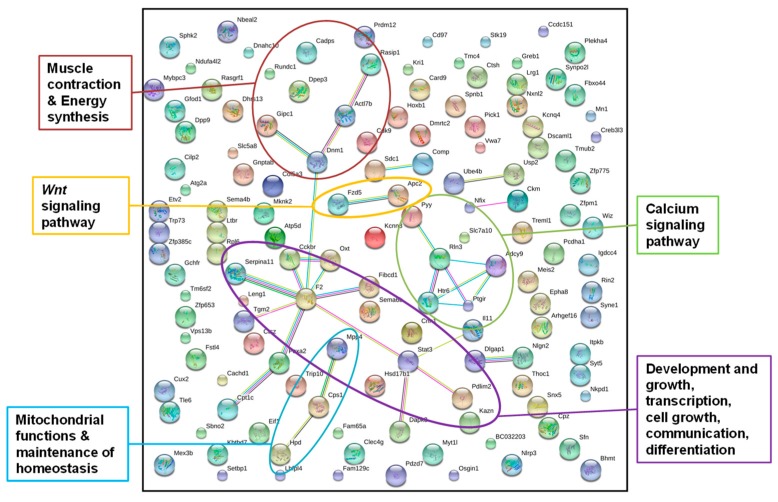
STRING gene network of genes from annotated DMRs in livers of F1 sons after paternal exposure to a low-protein diet; STRING gene network of genes from annotated DMRs detected in the livers of all five father-sorted son groups after paternal diet change (“experiment D”). The main metabolic pathways identified are labeled by colored circles (colors of dots were chosen by the STRING database and do not account for a certain gene function), and connections between dots indicate the interaction of gene products.

**Figure 4:**
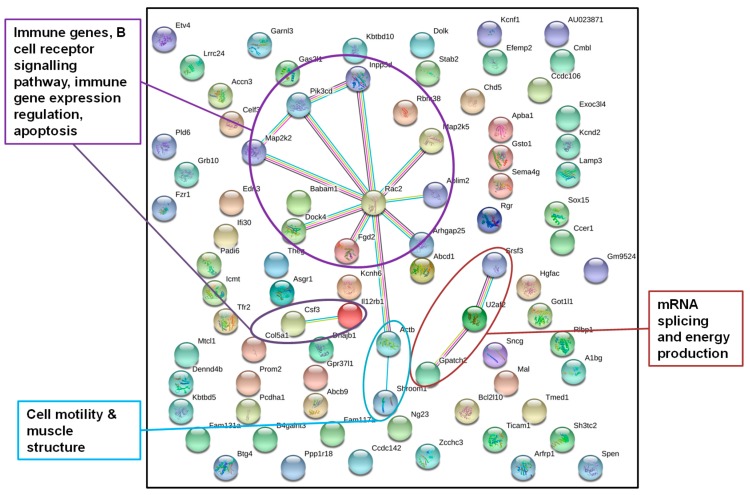
STRING gene network of genes from annotated DMRs in livers of F1 sons after paternal exposure to temperature increase; STRING gene network of genes from DMRs detected in the livers of at least four of the five father-sorted son groups after paternal exposure to increased temperature (“experiment H”). Gene network analysis identified genes encoding for proteins (dots) with a function in the immune system, cell structure, and RNA splicing (colors of dots were chosen by the STRING database and do not account for a certain gene function).

In this analysis, we combined *hypo*methylated and *hyper*methylated genes, because we aimed to identify all epigenetically affected pathways, irrespective of genes being activated or inhibited. We incorporated annotated DMRs overlapping with at least one protein-coding gene that were present in all father-sorted son groups for F1L_CD_ versus F1L_D_ in “experiment D” (*n_genes_* = 155, Figure 3, Table S1) and in at least four of the five father-sorted son groups for F1L_CH_ versus F1L_H_ in “experiment H” (*n_genes_* = 84, Figure 4, Table S2), and submitted them to the STRING database for gene network identification.

#### 3.2.1. Identification of Epigenetically Affected Pathways after Paternal Low-Protein Diet (“Experiment D”)

Out of the 155 genes, 135 were recognized by the STRING database using gene function data of *Mus musculus* as a reference (Figure 3). We increased the stringency interaction score from default setting 0.4 to 0.5. A higher stringency score leads to a reduction in the number of interactions detected, but those that are still detected have a stronger backup by the available data that the STRING database uses for pathway detection. Even though we increased the stringency of the interaction score, the network analysis resulted in significantly more interactions than would be expected in a random data set of similar size (*n_obs_* = 31, *n_exp_* = 18, *p* = 0.00318). This indicated that the proteins were at least partially biologically connected as a group. After “experiment D”, we pooled the liver DNAs of the sons, sorted by fathers to reduce sequencing costs and to facilitate data handling. Unfortunately, this prevented the analysis of individual-specific DMRs. Therefore, we chose the most conservative approach and only accepted annotated DMRs, which were detected in all five father-sorted son groups. The network identified genes in pathways with main metabolic functions such as development and growth, transcription, cell growth, communication, differentiation, muscle contraction and energy synthesis, a Wnt signaling pathway, a calcium signaling pathway, mitochondrial functions, and the maintenance of homeostasis.

#### 3.2.2. Identification of Epigenetically Affected Pathways after Paternal Exposure to a Temperature Increase (“Experiment H”)

DNA from sons (F1L_CH_, *n* = 16; F1L_H_, *n* = 18) was sequenced individually, wherefore we obtained individual-specific DMRs. To investigate a more general response to paternal heat exposure within this very detailed dataset, we focused on DMRs detected in at least four of the five father-sorted son groups. After paternal exposure to a temperature increase, 84 out of the 98 genes identified were also recognized by the STRING database, again using gene function data of *M. musculus* as a reference (Figure 4) and applying a stringency of 0.5. The network displayed significantly more interactions than expected by chance (*n_obs_* = 15, *n_exp_* = 6, *p* = 0.0029). For “experiment H”, the STRING network identified genes in pathways with immune functions, in B cell receptor signaling, in immune genes expression, in apoptosis, in cell structure formation, and in RNA splicing and energy production.

#### 3.2.3. Annotated Differentially Methylated Regions Shared in Response to Changes of Either One or Both Environmental Factors

**Figure 5:**
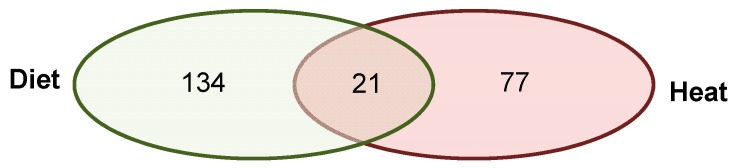
Venn diagram of genes with DMRs after both environmental factors. Of the 155 genes with DMRs identified in the livers of sons sired after a paternal protein diet and the 98 genes with DMRs identified after paternal heat exposure, 21 DMRs were shared between both experiments (diet and heat). Of those, three DMRs were overlapping promoter regions, and 18 DMRs were overlapping CDSes (listed in Table 1).

We detected 21 annotated DMRs that were shared in all five father-sorted son groups between the experiments (D vs H), of which 18 were located in CDS and three in promoter regions (Figure 5). When submitting these genes to STRING, the network analysis (again using a stringency score of 0.5) revealed no connections. We further investigated the genes’ function(s) using gene ontologies (Table 1). Among the 21 genes, one had an unknown function, whereas the 20 known genes were involved in male germ line development and spermatogenesis, immune response, growth factor activation and transcription activity, cell-to-cell signaling and cell fate decisions, calcium binding and response to stimulus, and ATP binding and mitochondrial muscle contraction.

### 3.3. Testing Hypotheses H1–H3

Our results rejected hypothesis H1 and H2, but not H3, because changes in the two environmental factors caused a “factor-specific” as well as a “general” response, represented by methylation changes that were targeted both at shared and at specific gene pathways.

## 4. Discussion

We here demonstrated that the paternally inherited epigenetic response of sons to changes in two environmental factors, a low-protein diet and a temperature increase, was composed of factor-specific methylation changes of genes with functions in the specific physiological response pathways (as hypothesized in H2), and of factor-unspecific methylation changes (as hypothesized in H1). Thus, our data supported hypothesis H3, the combination of both a specific and a general response.

Our results implicate that epigenetic modifications convey at least two types of environmental information to the subsequent generation(s): One is that the environment has changed (“alert information”), and the other is what factor has changed (“adequate response information”). This information provides increased adaptability to the offspring and a directed nonrandom epigenetic response. Thus, epigenetic modifications might be crucially important for (wild) mammal species to directly respond to and prepare offspring for potential environmental changes. By increasing phenotypic plasticity to intrinsic and extrinsic factors (even for offspring), epigenetic modifications close the gap between quick (and short-lasting) physiological responses and phenotypic shifts via very long-lasting mutational changes.

### 4.1. Specific and General Response

Most environmental epigenetic studies have been initiated for medical reasons to further our understanding of disease phenotypes (e.g., in response to environmental chemicals) [35,36]. Responses, phenotypes, and genes detected to be involved have been highly diverse and dependent on the chemical (Reference [36], Table 1 therein). In different studies using environmental chemicals (or pollutants) on lab rats, it has been shown that exposure to different chemicals caused different epigenetic responses (summarized in Table 1 in Reference [37]).

In contrast to our study, the above-mentioned studies on environmental toxins were either performed on inbred lab rat strains, human blood cells, or human cell cultures challenged with chemical compounds whose impact might vary depending on the degree of toxicity [38].

In our study, we applied two moderate and nonhazardous challenges to the guinea pigs. One was a 10 °C increase in ambient temperature (to 30 °C), and the other one was a 42% decrease in the protein content of their diet. These challenges represented environmental fluctuations, which guinea pigs may experience in their natural environment in South America, where they are widely distributed [27,28].

In contrast to the studies with hazardous chemicals mentioned above, in which researchers used genetically uniform model species, the wild guinea pigs in our study were genetically heterogeneous, with the potential for providing a wider “window” of response possibilities. This potential became even larger when considering that the wild guinea pig is a generalist species, living in a wide range of different habitats and along a great altitudinal gradient. As such, one would expect them to be responsive to a large variety of outer stimuli [27,28] and to be well-equipped with an epigenetic “tool box” to respond sufficiently to such a large variety of stimuli. A well-equipped “tool-box” increases the degree of plasticity of responses to outer stimuli, leading to more specific (better fine-tuned) epigenetic responses, which was what we observed in our experiment(s). The epigenetic mechanism might thus be crucial for wild species in globally changing environments.

In response to a paternal low-protein diet, DNA methylation patterns shifted in the livers of naïve sons (sired after the paternal low-protein diet, “experiment D”). The genes identified were in functional parts of pathways with main metabolic functions, such as development and growth, transcription, cell growth, communication, differentiation, muscle contraction and energy synthesis, Wnt signaling, calcium signaling, mitochondrial functions, and maintenance of homeostasis. Differential methylation in genes clustering within these pathways indicated an alteration in activation of these pathways, which caused an overall metabolic shift in response to the changed composition of the paternal diet in the sons. The Wnt signaling pathway, for example, reacts to outer stimuli through its Wnt (Wingless) and Integrator Complex Subunit 2 (Int-2) signal proteins [39]. These signals convey information to the organs triggering metabolic processes, which stabilize or regain homeostasis [40]. As the liver is the main metabolic organ, it stores energy in the form of glycogen, whose hydrolysis to glucose subsequently generates the energy required for a systemic response, represented in shifts in the metabolic pathways detected here.

In contrast, when fathers were exposed to a temperature increase, DNA methylation patterns and levels in the livers of naïve sons (sired after paternal exposure, “experiment H”) were changed in different genes (compared with the “low-protein diet response”, “experiment D”). These genes belonged to pathways responsible for immune function, B cell receptor signaling, immune gene expression, apoptosis, as well as in cell structure and RNA splicing and energy production. Clearly, these major pathways indicated a regulation of genes protecting or even improving the animals’ health. Exposure to heat induces apoptosis and DNA damage [41,42,43], affects nutritional, physiological, and reproductive functions, and can reduce spermatogenic activity.

Whether such a response is species-specific or can be generalized across rodents (or even mammals) remains to be seen, because epigenetic studies on other mammals exposed to a prolonged increase in ambient temperature have (to the best of our knowledge) not yet been performed.

The general response (genes with DMRs observed in both experiments) involved genes that were part of pathways with general functions such as immune-response (*Icam5*, *Mapk5)*, growth factor activation and transcription activity (*Med26*, *Dock6*, *Mmp9*, *Map3k6*, *Eps8l2)*, cell-to-cell signaling and cell fate decisions (*Notch4*, *Icam5*, *Hmnc2*, *Kcns1*), calcium binding and response to stimulus (*Hmnc2*), and ATP binding and mitochondrial muscle contraction (*Myh14*, *Dock6*). The latter two are genes involved in energy budgeting. Finding epigenetic modifications in those genes in both experiments was expected, because the liver stores energy in the form of glycogen, which is needed to provide energy to respond to environment changes. It also involved genes that were part of male-specific pathways such as male germ line development and spermatogenesis (*Svp*, *Sox13*, *Klhl10*), which might reflect the paternal (instead of the maternal) exposure. It is noteworthy that some DMRs might represent an age effect, because DNA methylation patterns change with age. To address this within the experimental setup, we narrowed the time window between the first and second mating to six months (the shortest period possible), in order to avoid reproductive stress on the females.

The liver is the main thermoregulatory and metabolic organ and therefore the representative organ necessary to adjust metabolism to outer stimuli in order to regain homeostasis. The comparison of the epigenetic responses in the livers of sons sired in the two experiments (experiments “D” and “H”) allowed for disentangling the response components responsible for “alert information” and “adequate response information”. In terms of moderate nutrition and temperature changes, adjustments in the energy budget going along with gene expression changes and changes in regulation were expected, as well as their effect on growth and the immune system.

It is noteworthy that while DMRs occur at identical genes, the expression may not be regulated in the same direction. For example, previously we have shown that the transcription factor *Stat3* was hypermethylated and repressed in sons sired after paternal heat exposure compared to control sons (F1L_CH_ vs F1L_H_) [29]. However, in sons sired after their fathers’ diet alteration, *Stat3* was also hypermethylated, but was higher expressed compared to control sons (F1L_CD_ vs F1L_D_). The diverging results for *Stat3* in experiments “D” and “H” also supported our “adequate response information” hypothesis, but they also showed that we still do not completely understand epigenetic regulatory mechanisms, because hypermethylation is associated with both up- and downregulation of gene expression. In the current study, we aimed to investigate the specific and general epigenetic response to two environmental factors within the same model. In order to investigate the adaptive value of those epigenetic changes, the impact on gene expression needs to be investigated on a transcriptomic level.

### 4.2. Alternative Explanation

Besides regulating gene expression (and its accompanying adaptive effects), the methylation of DNA is also a mechanism to silence transposable elements, preventing those elements from jumping within the genome, thereby causing genomic destabilization [44,45]. Malnutrition is known to lead to a shortage in methyl donors, which are needed by methyltransferases for the methylation of DNA. Thus, modifications in methylation patterns could be a mechanism to protect the DNA and its chromatin structure, to activate genomic parts that may act as chaperones for, e.g., protein degradation, such as heat shock proteins [46].

### 4.3. The Father’s Role: Roaming Males Increase Genetic and Epigenetic Diversity

In wild mammal species, males predominantly disperse, while females mostly stay at their natal site (philopatry). Dispersing males are seen as carriers of gene-flow across populations and thus maintain genetic diversity [47]. Our results and those of other studies [7,48,49,50] imply that dispersing males might also increase epigenetic diversity in populations they immigrate into. The findings presented here not only show the high relevance of paternal epigenetic inheritance and thus the importance of the father in adaptation processes, but also that the pattern of paternal epigenetic inheritance highly depends on the environmental factor that has changed.

### 4.4. Evolutionary Consequences of Epigenetic Inheritance

Epigenetically mediated regulation may increase genotypically encoded adaptive plasticity to a widened epigenetically modified phenotypic plasticity. An epigenetic response may prepare subsequent generations for novel conditions by providing an additional layer of shorter-term adaptations as a rapid but nonrandom reaction to outer changes. That way, wild mammals are well prepared to cope with climate and vegetation changes in their niche without having to depend only on random genomic mutations. If the novel outer condition persists, flexible epigenetic changes might later-on be stably incorporated into the genome for long-term genomic adaptation.

## 5. Conclusions

Two types of environmental information, “alert information” and “adequate response information”, seem to be transmitted to the subsequent generation(s), potentially increasing adaptability of the offspring and a directed nonrandom epigenetic response to specific environmental factors. This indicates the importance of epigenetic patterns for (wild) mammal species to directly respond to and prepare offspring for potential environmental changes. Furthermore, this environmental-specific response in the same functional organ is an important finding when working with free-ranging wild species (e.g., in diverse habitats, migration, and urbanization) to discriminate among environmental effects. We hope that our data will enable other wildlife researchers to differentiate among these potential effects within their species, and that it is important to be aware of these.

## Figures and Tables

**Table 1:** Twenty-one genes with DMRs shared in all five father-sorted son groups of the “nutrition” and “heat” experiments.

Gene Name (Ensembl ID)	Full Gene Name	Regulatory Region	Gene Ontology (GO) Term
*Dock6* (ENSCPOG00000026620)	Dedicator of Cytokinesis 6	CDS	Positive regulation of hydrolyze guanosine triphosphate (GTP) GTPase activity, guanyl-nucleotide exchange factor activity, small GTPase-mediated signal transduction, cytoplasm
*Eps8l2* (ENSCPOG00000003802)	Epidermal growth factor receptor kinase substrate 8-like protein 2	CDS	Cytoplasm, plasma membrane, protein complex, protein binding, positive regulation of GTPase activity, actin binding, extracellular exosome, Rho guanyl-nucleotide exchange factor activity, regulation of Rho protein signal transduction, actin filament binding, Rho protein signal transduction, ruffle, ruffle membrane, vesicle, Rac guanyl-nucleotide exchange factor activity, Rac protein signal transduction, positive regulation of ruffle assembly
*Hmcn2* (ENSCPOG00000013220)	Hemicentin 2	CDS	Calcium ion binding, protein binding, cell junction, cell cortex, response to stimulus
*Icam5* (ENSCPOG00000000999)	Intercellular adhesion molecule 5	CDS	Plasma membrane, integral component of plasma membrane, protein binding, single organismal cell-cell adhesion cell adhesion, phagocytosis, integrin binding
*Kcns1* (ENSCPOG00000013646)	Potassium voltage-gated channel subfamily S member 1	CDS	Transmembrane transport, protein homo-oligomerization, perinuclear region of cytoplasm, protein binding, membrane voltage-gated potassium channel complex, potassium ion transmembrane transport, potassium ion transport, ion transport, ion channel activity, delayed rectifier potassium channel activity, regulation of delayed rectifier potassium channel activity, potassium channel regulator activity
*Klhl10* (ENSCPOG00000002818)	Kelch-Like Family Member 10, Testicular Tissue Protein Li 104	CDS	Cytoplasm, homeostasis of number of cells within a tissue, protein binding, ubiquitin-protein transferase activity, protein ubiquitination, spermatid development, Cul3-RING ubiquitin ligase complex, cell morphogenesis, male gonad development, fertilization, male genitalia morphogenesis
*Map3k6* (ENSCPOG00000012359)	Mitogen-Activated Protein Kinase 6	CDS	Protein kinase activity, ATP binding, protein phosphorylation, MAP kinase, kinase activity, activation of MAPKK activity, magnesium ion binding
*Mmp9* (ENSCPOG00000007559)	Matrix Metallopeptidase 9	CDS	Proteolysis, metalloendopeptidase activity, ossification, collagen catabolic process, leukocyte migration
*Myh14* (ENSCPOG00000002223)	Myosin-14	CDS	ATP binding, metabolic process, protein binding, actin filament binding, sensory perception of sound, calmodulin binding, actomyosin structure organization, neuronal action potential, axon, motor activity, myosin complex, regulation of cell shape, stress fiber, skeletal muscle tissue development, vocalization behavior, skeletal muscle contraction, mitochondrion morphogenesis, actin-dependent ATPase activity, actin filament-based movement, microfilament motor activity, actomyosin, myosin filament
*Notch4* (ENSCPOG00000000591)	Neurogenic locus notch homolog 4	CDS	Integral component of membrane, calcium ion binding, protein binding, multicellular organismal development, cell differentiation, Notch signaling pathway, regulation of developmental process, mammary gland development, endothelial cell differentiation, endothelial cell morphogenesis
*Otud6a* (ENSCPOG00000012916)	OTU domain containing 6	CDS	Skeletal system morphogenesis
*Pclo* (ENSCPOG00000009376)	Piccolo Presynaptic Cytomatrix Protein	CDS	Calcium ion binding, intracellular, metal ion binding, protein binding, cell junction, presynaptic active zone, synapse assembly, cytoskeleton organization, insulin secretion, extracellular exosome, calcium-dependent phospholipid binding, regulation of exocytosis, synapse, postsynaptic density, cAMP-mediated signaling, profilin binding, synaptic vesicle targeting
*Plekhh3* (ENSCPOG00000010080)	Pleckstrin Homology, MyTH4 And FERM Domain Containing H3	CDS	Signal transduction, cytoskeleton
*Sh3gl1* (ENSCPOG00000023236)	SH3 Domain Containing GRB2 Like 1, Endophilin A2	CDS	Cytoplasm, protein binding, cell junction, early endosome, membrane, identical protein binding, lipid binding, endocytosis, podosome, cell projection, phosphatase binding, GTPase binding
*Sigirr* (ENSCPOG00000025549)	Single Immunoglobulin and Toll-Interleukin 1 Receptor (TIR) Domain	CDS	Integral component of membrane, signal transduction, protein binding, membrane, negative regulation of cytokine-mediated signaling pathway, acute-phase response, negative regulation of sequence-specific DNA binding transcription factor activity, negative regulation of chemokine biosynthetic process
*Slc46a2* (ENSCPOG00000001823)	Solute Carrier Family 46 Member 2	CDS	Integral component of membrane, transmembrane transport, molecular function, plasma membrane, cell surface, transporter activity, T cell homeostasis, regulation of T cell differentiation, negative regulation of T cell apoptotic process, thymus development
*Sox13* (ENSCPOG00000006604)		CDS	Nucleus, regulation of transcription, DNA-templated, sequence-specific DNA binding
*Med26* (ENSCPOG00000008968)	Mediator complex subunit 26 (adopted from mouse)	CDS	Nucleus, DNA binding, transcription, DNA-templated, regulation of transcription from RNA polymerase II promoter, transcription initiation from RNA polymerase II promoter, nucleoplasm RNA polymerase II transcription cofactor activity, mediator complex, transcription coactivator activity
*Sncg* (ENSCPOG00000023463)	Synuclein Gamma	Promoter	Cytoplasm, synaptic transmission, perinuclear region of cytoplasm, protein binding, synapse organization, extracellular exosome, microtubule organizing center, axon, neuronal cell body spindle, adult locomotory behavior, protein secretion, regulation of neurotransmitter secretion, regulation of dopamine secretion
*Svp-1* (ENSCPOG00000025237)	Seminal vesicle polypeptide	Promoter	Copulation, DNA binding, transcription
Unknown (ENSCPOG00000022766)		Promoter	

DMR: DNA methylation changes; CDS: coding sequence.

## Supplementary Materials

The following are available online at http://www.mdpi.com/2073-4425/10/1/4/s1, Link S1: Data accessibility statement; Table S1: List of annotated DMRs in “experiment D” present in all father-sorted son groups (F1L_C_ vs F1L_D_) with at least one annotated gene of protein-coding genes (applied in Figure 3); Table S2: Annotated DMRs in “experiment H” present in at least four of five father-sorted son groups (F1L_C_ vs F1L_H_) with at least one annotated gene of protein-coding genes present (applied in Figure 4).
