# Supplementary material for: Environmental Change-Dependent Inherited Epigenetic Response
Source: Genes (Basel). 2018 Dec 21;10(1):4. doi: 10.3390/genes10010004 (PMC6356568; doi:10.3390/genes10010004)
Supplement: Supplementary file 1 [file genes-10-00004-s001.docx]

**Supplementary Materials**

**Link S1.** Data accessibility statement.

Next-generation sequencing data were uploaded to the National Center for Biotechnology Information Short Reads Archive (http://www.ncbi.nim.nih.gov/sra) and are publicly accessible under the SRA study accession number SRP048942 in fastq file format. Raw data, coverage, methylation ratios, and DMR tables are accessible on Dryad (http://dx.doi.org/10.5061/dryad.0f8q1).

**Table S1.** List of annotated DMRs in “experiment D” present in all father-sorted son groups (F1L_C_ vs F1L_D_) with at least one annotated gene of protein-coding genes (applied in Figure 3).

| **ID** | **Gene Name** | **Annotation Type** | **No. of Father-Son Groups** |
| --- | --- | --- | --- |
| ENSCPOG00000002019_tss | *CTSH* | tss | 5 |
| ENSCPOG00000024328_tss | *DAPK3* | tss | 5 |
| ENSCPOG00000026058_promoter | *CAPS* | promoter | 5 |
| ENSCPOG00000009855_promoter | *CNN1* | promoter | 5 |
| ENSCPOG00000010134_promoter | *CREB3L3* | promoter | 5 |
| ENSCPOG00000002019_promoter | *CTSH* | promoter | 5 |
| ENSCPOG00000024328_promoter | *DAPK3* | promoter | 5 |
| ENSCPOG00000007466_promoter | *DHRS13* | promoter | 5 |
| ENSCPOG00000001805_promoter | *DMRTC2* | promoter | 5 |
| ENSCPOG00000005908_promoter | *DPEP3* | promoter | 5 |
| ENSCPOG00000023894_promoter | *DPP9* | promoter | 5 |
| ENSCPOG00000014350_promoter | *EIF1* | promoter | 5 |
| ENSCPOG00000004238_promoter | *FAM65A* | promoter | 5 |
| ENSCPOG00000027068_promoter | *FOXA2* | promoter | 5 |
| ENSCPOG00000014109_promoter | *GCHFR* | promoter | 5 |
| ENSCPOG00000022501_promoter | *GNPTAB* | promoter | 5 |
| ENSCPOG00000022183_promoter | *HOXB1* | promoter | 5 |
| ENSCPOG00000000195_promoter | *HPD* | promoter | 5 |
| ENSCPOG00000004971_promoter | *KCNQ4* | promoter | 5 |
| ENSCPOG00000027247_promoter | *LENG1* | promoter | 5 |
| ENSCPOG00000014879_promoter | *MKNK2* | promoter | 5 |
| ENSCPOG00000013368_promoter | *MYBPC3* | promoter | 5 |
| ENSCPOG00000013526_promoter | *Rpl6* | promoter | 5 |
| ENSCPOG00000022916_promoter | *Tm6sf2* | promoter | 5 |
| ENSCPOG00000023182_promoter | unknown gene | promoter | 5 |
| ENSCPOG00000025126_promoter | unknown gene | promoter | 5 |
| ENSCPOG00000025413_promoter | unknown gene | promoter | 5 |
| ENSCPOG00000025944_promoter | unknown gene | promoter | 5 |
| ENSCPOG00000027136_promoter | unknown gene | promoter | 5 |
| ENSCPOG00000012842_promoter | *NDUFA4L2* | promoter | 5 |
| ENSCPOG00000024344_promoter | *NXNL2* | promoter | 5 |
| ENSCPOG00000006612_promoter | *OSGIN1* | promoter | 5 |
| ENSCPOG00000005901_promoter | *PRDM12* | promoter | 5 |
| ENSCPOG00000023437_promoter | *PYY* | promoter | 5 |
| ENSCPOG00000006803_promoter | *RUNDC1* | promoter | 5 |
| ENSCPOG00000008875_promoter | *SDC1* | promoter | 5 |
| ENSCPOG00000007545_promoter | *SERPINA11* | promoter | 5 |
| ENSCPOG00000010055_promoter | *SLC5A8* | promoter | 5 |
| ENSCPOG00000013715_promoter | *SLC7A10* | promoter | 5 |
| ENSCPOG00000007908_promoter | *SNX5* | promoter | 5 |
| ENSCPOG00000009084_promoter | *SYT5* | promoter | 5 |
| ENSCPOG00000000959_promoter | *THOC1* | promoter | 5 |
| ENSCPOG00000026363_promoter | *TLE6* | promoter | 5 |
| ENSCPOG00000004111_promoter | *TREML1* | promoter | 5 |
| ENSCPOG00000015429_promoter | *UBE4B* | promoter | 5 |
| ENSCPOG00000009735_promoter | *WIZ* | promoter | 5 |
| ENSCPOG00000006417_promoter | *ZNF385C* | promoter | 5 |
| ENSCPOG00000002019_start_codon | *CTSH* | codon | 5 |
| ENSCPOG00000024328_start_codon | *DAPK3* | codon | 5 |
| ENSCPOG00000005904_CDS | *ACTL7B* | CDS | 5 |
| ENSCPOG00000019749_CDS | *ADCY9* | CDS | 5 |
| ENSCPOG00000021628_CDS | *APC2* | CDS | 5 |
| ENSCPOG00000023427_CDS | *ARHGEF16* | CDS | 5 |
| ENSCPOG00000008573_CDS | *ATG2A* | CDS | 5 |
| ENSCPOG00000020167_CDS | *ATP5D* | CDS | 5 |
| ENSCPOG00000002532_CDS | *BHMT* | CDS | 5 |
| ENSCPOG00000025508_CDS | *C19orf38* | CDS | 5 |
| ENSCPOG00000021346_CDS | *C20orf72* | CDS | 5 |
| ENSCPOG00000011700_CDS | *CACHD1* | CDS | 5 |
| ENSCPOG00000015152_CDS | *CARD9* | CDS | 5 |
| ENSCPOG00000025979_CDS | *CCDC151* | CDS | 5 |
| ENSCPOG00000025284_CDS | *CCKBR* | CDS | 5 |
| ENSCPOG00000006388_CDS | *CD97* | CDS | 5 |
| ENSCPOG00000007219_CDS | *CILP2* | CDS | 5 |
| ENSCPOG00000019609_CDS | *CKM* | CDS | 5 |
| ENSCPOG00000013185_CDS | *CLEC4G* | CDS | 5 |
| ENSCPOG00000024360_CDS | *COL5A3* | CDS | 5 |
| ENSCPOG00000025029_CDS | *COMP* | CDS | 5 |
| ENSCPOG00000004146_CDS | *CPS1* | CDS | 5 |
| ENSCPOG00000015483_CDS | *CPT1C* | CDS | 5 |
| ENSCPOG00000025982_CDS | *CPZ* | CDS | 5 |
| ENSCPOG00000002019_CDS | *CTSH* | CDS | 5 |
| ENSCPOG00000026091_CDS | *CTSZ* | CDS | 5 |
| ENSCPOG00000020483_CDS | *CUX2* | CDS | 5 |
| ENSCPOG00000024328_CDS | *DAPK3* | CDS | 5 |
| ENSCPOG00000006550_CDS | *DLGAP1* | CDS | 5 |
| ENSCPOG00000013914_CDS | *DNAH10* | CDS | 5 |
| ENSCPOG00000022899_CDS | *DNM1* | CDS | 5 |
| ENSCPOG00000014040_CDS | *DSCAML1* | CDS | 5 |
| ENSCPOG00000021134_CDS | *EPHA8* | CDS | 5 |
| ENSCPOG00000013209_CDS | *ETV2* | CDS | 5 |
| ENSCPOG00000026682_CDS | *F2* | CDS | 5 |
| ENSCPOG00000013004_CDS | *FAM129C* | CDS | 5 |
| ENSCPOG00000026240_CDS | *FBXO44* | CDS | 5 |
| ENSCPOG00000006219_CDS | *FIBCD1* | CDS | 5 |
| ENSCPOG00000007594_CDS | *FSTL4* | CDS | 5 |
| ENSCPOG00000004116_CDS | *FZD5* | CDS | 5 |
| ENSCPOG00000003570_CDS | *GFOD1* | CDS | 5 |
| ENSCPOG00000022424_CDS | *GIPC1* | CDS | 5 |
| ENSCPOG00000006588_CDS | *GREB1* | CDS | 5 |
| ENSCPOG00000021970_CDS | *HSD17B1* | CDS | 5 |
| ENSCPOG00000001972_CDS | *HTR6* | CDS | 5 |
| ENSCPOG00000004519_CDS | *IGDCC4* | CDS | 5 |
| ENSCPOG00000009123_CDS | *IL11* | CDS | 5 |
| ENSCPOG00000013483_CDS | *ITPKB* | CDS | 5 |
| ENSCPOG00000007591_CDS | *KAZN* | CDS | 5 |
| ENSCPOG00000015718_CDS | *KCNN3* | CDS | 5 |
| ENSCPOG00000005531_CDS | *KIAA0240* | CDS | 5 |
| ENSCPOG00000010323_CDS | *KRI1* | CDS | 5 |
| ENSCPOG00000001556_CDS | *LHFPL4* | CDS | 5 |
| ENSCPOG00000002690_CDS | *LRG1* | CDS | 5 |
| ENSCPOG00000015661_CDS | *LTBR* | CDS | 5 |
| ENSCPOG00000015087_CDS | *MEIS2* | CDS | 5 |
| ENSCPOG00000008241_CDS | *MEX3B* | CDS | 5 |
| ENSCPOG00000003430_CDS | *MN1* | CDS | 5 |
| ENSCPOG00000014262_CDS | *MPP4* | CDS | 5 |
| ENSCPOG00000013246_CDS | *MYT1L* | CDS | 5 |
| ENSCPOG00000000707_CDS | *CCDC94* | CDS | 5 |
| ENSCPOG00000008898_CDS | *Kbtbd7* | CDS | 5 |
| ENSCPOG00000008920_CDS | unknown gene | CDS | 5 |
| ENSCPOG00000010359_CDS | unknown gene | CDS | 5 |
| ENSCPOG00000010842_CDS | *Znf653* | CDS | 5 |
| ENSCPOG00000012761_CDS | unknown gene | CDS | 5 |
| ENSCPOG00000020451_CDS | *Stk19* | CDS | 5 |
| ENSCPOG00000023481_CDS | unknown gene | CDS | 5 |
| ENSCPOG00000008167_CDS | *NBEAL2* | CDS | 5 |
| ENSCPOG00000022563_CDS | *NFIX* | CDS | 5 |
| ENSCPOG00000026614_CDS | *NKPD1* | CDS | 5 |
| ENSCPOG00000019317_CDS | *NLGN2* | CDS | 5 |
| ENSCPOG00000007681_CDS | *NLRP3* | CDS | 5 |
| ENSCPOG00000001717_CDS | *OXT* | CDS | 5 |
| ENSCPOG00000010618_CDS | *PCDHA1* | CDS | 5 |
| ENSCPOG00000020871_CDS | *PDLIM2* | CDS | 5 |
| ENSCPOG00000000952_CDS | *PDZD7* | CDS | 5 |
| ENSCPOG00000019992_CDS | *PGBD2* | CDS | 5 |
| ENSCPOG00000010802_CDS | *PICK1* | CDS | 5 |
| ENSCPOG00000008161_CDS | *PLEKHA4* | CDS | 5 |
| ENSCPOG00000001613_CDS | *PTGIR* | CDS | 5 |
| ENSCPOG00000005363_CDS | *RASGRF1* | CDS | 5 |
| ENSCPOG00000001881_CDS | *RASIP1* | CDS | 5 |
| ENSCPOG00000013179_CDS | *RIN2* | CDS | 5 |
| ENSCPOG00000013564_CDS | *RLN3* | CDS | 5 |
| ENSCPOG00000007477_CDS | *SBNO2* | CDS | 5 |
| ENSCPOG00000009429_CDS | *SEMA4B* | CDS | 5 |
| ENSCPOG00000002693_CDS | *SEMA6B* | CDS | 5 |
| ENSCPOG00000011164_CDS | *SETBP1* | CDS | 5 |
| ENSCPOG00000010401_CDS | *SFN* | CDS | 5 |
| ENSCPOG00000012734_CDS | *SPHK2* | CDS | 5 |
| ENSCPOG00000025923_CDS | *SPTB* | CDS | 5 |
| ENSCPOG00000007939_CDS | *STAT3* | CDS | 5 |
| ENSCPOG00000008325_CDS | *SYNE1* | CDS | 5 |
| ENSCPOG00000022018_CDS | *SYNPO2L* | CDS | 5 |
| ENSCPOG00000011896_CDS | *Tgm2* | CDS | 5 |
| ENSCPOG00000024120_CDS | *TMC4* | CDS | 5 |
| ENSCPOG00000007784_CDS | *TMUB2* | CDS | 5 |
| ENSCPOG00000010908_CDS | *TP73* | CDS | 5 |
| ENSCPOG00000014627_CDS | *TRIP10* | CDS | 5 |
| ENSCPOG00000001380_CDS | *TSPYL5* | CDS | 5 |
| ENSCPOG00000004199_CDS | unknown gene | CDS | 5 |
| ENSCPOG00000004520_CDS | *USP2* | CDS | 5 |
| ENSCPOG00000014447_CDS | *VPS13B* | CDS | 5 |
| ENSCPOG00000000869_CDS | *VWA7* | CDS | 5 |
| ENSCPOG00000024281_CDS | *ZFPM1* | CDS | 5 |
| ENSCPOG00000006417_CDS | *ZNF385C* | CDS | 5 |
| ENSCPOG00000022680_CDS | *ZNF775* | CDS | 5 |
| CpG_Island_scaffold0_1337070_1338019 | NA | CGI | 5 |
| CpG_Island_scaffold0_45575108_45575794 | NA | CGI | 5 |
| CpG_Island_scaffold0_51917148_51917761 | NA | CGI | 5 |
| CpG_Island_scaffold0_5444829_5445337 | NA | CGI | 5 |
| CpG_Island_scaffold0_6132346_6132845 | NA | CGI | 5 |
| CpG_Island_scaffold101_4818265_4819001 | NA | CGI | 5 |
| CpG_Island_scaffold102_2178091_2178635 | NA | CGI | 5 |
| CpG_Island_scaffold10_12845709_12846208 | NA | CGI | 5 |
| CpG_Island_scaffold10_17196688_17197665 | NA | CGI | 5 |
| CpG_Island_scaffold10_22855383_22856374 | NA | CGI | 5 |
| CpG_Island_scaffold10_31449750_31450536 | NA | CGI | 5 |
| CpG_Island_scaffold111_3791048_3791547 | NA | CGI | 5 |
| CpG_Island_scaffold111_897989_898494 | NA | CGI | 5 |
| CpG_Island_scaffold111_967414_968866 | NA | CGI | 5 |
| CpG_Island_scaffold115_134129_135216 | NA | CGI | 5 |
| CpG_Island_scaffold115_2179090_2179642 | NA | CGI | 5 |
| CpG_Island_scaffold115_3998488_3999011 | NA | CGI | 5 |
| CpG_Island_scaffold1183_17394_18264 | NA | CGI | 5 |
| CpG_Island_scaffold119_1622531_1623035 | NA | CGI | 5 |
| CpG_Island_scaffold119_1625761_1626514 | NA | CGI | 5 |
| CpG_Island_scaffold125_2800185_2801182 | NA | CGI | 5 |
| CpG_Island_scaffold12_21363230_21363729 | NA | CGI | 5 |
| CpG_Island_scaffold13_41290765_41292045 | NA | CGI | 5 |
| CpG_Island_scaffold140_872847_873353 | NA | CGI | 5 |
| CpG_Island_scaffold1428_6585_8912 | NA | CGI | 5 |
| CpG_Island_scaffold144_116651_117388 | NA | CGI | 5 |
| CpG_Island_scaffold14_19969216_19969805 | NA | CGI | 5 |
| CpG_Island_scaffold154_131870_132428 | NA | CGI | 5 |
| CpG_Island_scaffold155_2091335_2092331 | NA | CGI | 5 |
| CpG_Island_scaffold156_1107625_1108615 | NA | CGI | 5 |
| CpG_Island_scaffold156_1269767_1270398 | NA | CGI | 5 |
| CpG_Island_scaffold159_427506_428175 | NA | CGI | 5 |
| CpG_Island_scaffold159_607250_608357 | NA | CGI | 5 |
| CpG_Island_scaffold15_18211918_18212731 | NA | CGI | 5 |
| CpG_Island_scaffold15_31530860_31531427 | NA | CGI | 5 |
| CpG_Island_scaffold15_31631386_31631915 | NA | CGI | 5 |
| CpG_Island_scaffold15_34216137_34217078 | NA | CGI | 5 |
| CpG_Island_scaffold15_41051785_41052658 | NA | CGI | 5 |
| CpG_Island_scaffold160_1952772_1953271 | NA | CGI | 5 |
| CpG_Island_scaffold160_2025783_2026768 | NA | CGI | 5 |
| CpG_Island_scaffold167_328239_329585 | NA | CGI | 5 |
| CpG_Island_scaffold168_1617076_1617588 | NA | CGI | 5 |
| CpG_Island_scaffold17_6161759_6162376 | NA | CGI | 5 |
| CpG_Island_scaffold181_48758_49384 | NA | CGI | 5 |
| CpG_Island_scaffold183_1455065_1455629 | NA | CGI | 5 |
| CpG_Island_scaffold184_940996_941687 | NA | CGI | 5 |
| CpG_Island_scaffold186_243865_244396 | NA | CGI | 5 |
| CpG_Island_scaffold187_115459_115958 | NA | CGI | 5 |
| CpG_Island_scaffold189_845122_845656 | NA | CGI | 5 |
| CpG_Island_scaffold18_1391509_1392145 | NA | CGI | 5 |
| CpG_Island_scaffold18_2748973_2749481 | NA | CGI | 5 |
| CpG_Island_scaffold18_38954877_38955433 | NA | CGI | 5 |
| CpG_Island_scaffold18_661068_662065 | NA | CGI | 5 |
| CpG_Island_scaffold18_8371089_8371639 | NA | CGI | 5 |
| CpG_Island_scaffold195_686680_687304 | NA | CGI | 5 |
| CpG_Island_scaffold195_715731_716232 | NA | CGI | 5 |
| CpG_Island_scaffold1_23540202_23541659 | NA | CGI | 5 |
| CpG_Island_scaffold1_25231484_25232087 | NA | CGI | 5 |
| CpG_Island_scaffold1_65977794_65978780 | NA | CGI | 5 |
| CpG_Island_scaffold1_75146902_75147546 | NA | CGI | 5 |
| CpG_Island_scaffold1_77915177_77915676 | NA | CGI | 5 |
| CpG_Island_scaffold1_79119507_79120353 | NA | CGI | 5 |
| CpG_Island_scaffold203_368081_368954 | NA | CGI | 5 |
| CpG_Island_scaffold203_442790_443329 | NA | CGI | 5 |
| CpG_Island_scaffold2081_1_2385 | NA | CGI | 5 |
| CpG_Island_scaffold2081_2386_3587 | NA | CGI | 5 |
| CpG_Island_scaffold20_10032646_10033145 | NA | CGI | 5 |
| CpG_Island_scaffold20_18916093_18916681 | NA | CGI | 5 |
| CpG_Island_scaffold20_5581381_5581889 | NA | CGI | 5 |
| CpG_Island_scaffold216_527627_528137 | NA | CGI | 5 |
| CpG_Island_scaffold226_36753_37440 | NA | CGI | 5 |
| CpG_Island_scaffold226_367983_368725 | NA | CGI | 5 |
| CpG_Island_scaffold226_526774_527277 | NA | CGI | 5 |
| CpG_Island_scaffold226_668007_668506 | NA | CGI | 5 |
| CpG_Island_scaffold22_8670977_8671537 | NA | CGI | 5 |
| CpG_Island_scaffold22_8684047_8684858 | NA | CGI | 5 |
| CpG_Island_scaffold22_9410033_9410915 | NA | CGI | 5 |
| CpG_Island_scaffold237_64188_64940 | NA | CGI | 5 |
| CpG_Island_scaffold239_276667_277259 | NA | CGI | 5 |
| CpG_Island_scaffold23_20634325_20634933 | NA | CGI | 5 |
| CpG_Island_scaffold25_15523908_15524804 | NA | CGI | 5 |
| CpG_Island_scaffold25_17018037_17018609 | NA | CGI | 5 |
| CpG_Island_scaffold25_24938618_24939445 | NA | CGI | 5 |
| CpG_Island_scaffold25_25074653_25075851 | NA | CGI | 5 |
| CpG_Island_scaffold25_26162694_26164166 | NA | CGI | 5 |
| CpG_Island_scaffold25_26171625_26172999 | NA | CGI | 5 |
| CpG_Island_scaffold25_26173207_26173835 | NA | CGI | 5 |
| CpG_Island_scaffold25_26176251_26177642 | NA | CGI | 5 |
| CpG_Island_scaffold25_28094817_28095323 | NA | CGI | 5 |
| CpG_Island_scaffold25_28180968_28181488 | NA | CGI | 5 |
| CpG_Island_scaffold25_7195910_7196447 | NA | CGI | 5 |
| CpG_Island_scaffold25_9300148_9300650 | NA | CGI | 5 |
| CpG_Island_scaffold27_15967211_15967777 | NA | CGI | 5 |
| CpG_Island_scaffold27_2205063_2205931 | NA | CGI | 5 |
| CpG_Island_scaffold27_2214546_2215102 | NA | CGI | 5 |
| CpG_Island_scaffold27_2288380_2288928 | NA | CGI | 5 |
| CpG_Island_scaffold27_2662972_2663865 | NA | CGI | 5 |
| CpG_Island_scaffold283_85083_85676 | NA | CGI | 5 |
| CpG_Island_scaffold28_21714907_21715502 | NA | CGI | 5 |
| CpG_Island_scaffold28_22426447_22427441 | NA | CGI | 5 |
| CpG_Island_scaffold28_26510199_26510752 | NA | CGI | 5 |
| CpG_Island_scaffold309_47727_48229 | NA | CGI | 5 |
| CpG_Island_scaffold320_108027_108527 | NA | CGI | 5 |
| CpG_Island_scaffold320_26625_27293 | NA | CGI | 5 |
| CpG_Island_scaffold320_39632_40268 | NA | CGI | 5 |
| CpG_Island_scaffold32_21720635_21721169 | NA | CGI | 5 |
| CpG_Island_scaffold334_100339_101019 | NA | CGI | 5 |
| CpG_Island_scaffold334_104682_105385 | NA | CGI | 5 |
| CpG_Island_scaffold368_162206_162736 | NA | CGI | 5 |
| CpG_Island_scaffold382_207067_207727 | NA | CGI | 5 |
| CpG_Island_scaffold38_4489920_4490424 | NA | CGI | 5 |
| CpG_Island_scaffold38_502693_503521 | NA | CGI | 5 |
| CpG_Island_scaffold38_9125934_9126944 | NA | CGI | 5 |
| CpG_Island_scaffold39_1944564_1945124 | NA | CGI | 5 |
| CpG_Island_scaffold39_4272255_4272781 | NA | CGI | 5 |
| CpG_Island_scaffold429_156919_158190 | NA | CGI | 5 |
| CpG_Island_scaffold42_12821259_12822397 | NA | CGI | 5 |
| CpG_Island_scaffold42_13254013_13254948 | NA | CGI | 5 |
| CpG_Island_scaffold42_14674223_14674979 | NA | CGI | 5 |
| CpG_Island_scaffold42_2809881_2811011 | NA | CGI | 5 |
| CpG_Island_scaffold42_6229646_6230473 | NA | CGI | 5 |
| CpG_Island_scaffold42_6305294_6305858 | NA | CGI | 5 |
| CpG_Island_scaffold43_10813492_10814436 | NA | CGI | 5 |
| CpG_Island_scaffold43_3286590_3287153 | NA | CGI | 5 |
| CpG_Island_scaffold43_7998279_7998778 | NA | CGI | 5 |
| CpG_Island_scaffold45_12477236_12477799 | NA | CGI | 5 |
| CpG_Island_scaffold4_15575264_15575763 | NA | CGI | 5 |
| CpG_Island_scaffold4_20149964_20150996 | NA | CGI | 5 |
| CpG_Island_scaffold4_20683303_20683832 | NA | CGI | 5 |
| CpG_Island_scaffold4_825762_826653 | NA | CGI | 5 |
| CpG_Island_scaffold509_57172_57700 | NA | CGI | 5 |
| CpG_Island_scaffold511_88878_89377 | NA | CGI | 5 |
| CpG_Island_scaffold51_11354749_11355603 | NA | CGI | 5 |
| CpG_Island_scaffold537_1725_8209 | NA | CGI | 5 |
| CpG_Island_scaffold537_1_1720 | NA | CGI | 5 |
| CpG_Island_scaffold53_12038211_12038771 | NA | CGI | 5 |
| CpG_Island_scaffold53_12277552_12278398 | NA | CGI | 5 |
| CpG_Island_scaffold53_12734240_12734860 | NA | CGI | 5 |
| CpG_Island_scaffold53_8764673_8765172 | NA | CGI | 5 |
| CpG_Island_scaffold53_9333359_9333895 | NA | CGI | 5 |
| CpG_Island_scaffold53_9791279_9791919 | NA | CGI | 5 |
| CpG_Island_scaffold54_4729730_4730763 | NA | CGI | 5 |
| CpG_Island_scaffold56_993960_994677 | NA | CGI | 5 |
| CpG_Island_scaffold574_83082_83652 | NA | CGI | 5 |
| CpG_Island_scaffold5_18627344_18628045 | NA | CGI | 5 |
| CpG_Island_scaffold61_9230212_9230749 | NA | CGI | 5 |
| CpG_Island_scaffold62_1122935_1123461 | NA | CGI | 5 |
| CpG_Island_scaffold63_7395429_7396044 | NA | CGI | 5 |
| CpG_Island_scaffold63_9440947_9441602 | NA | CGI | 5 |
| CpG_Island_scaffold653_57469_58078 | NA | CGI | 5 |
| CpG_Island_scaffold67_7188249_7188771 | NA | CGI | 5 |
| CpG_Island_scaffold67_8688910_8689510 | NA | CGI | 5 |
| CpG_Island_scaffold67_9557580_9558116 | NA | CGI | 5 |
| CpG_Island_scaffold687_54045_54964 | NA | CGI | 5 |
| CpG_Island_scaffold6_27382255_27382982 | NA | CGI | 5 |
| CpG_Island_scaffold6_3123065_3124006 | NA | CGI | 5 |
| CpG_Island_scaffold6_3163589_3164108 | NA | CGI | 5 |
| CpG_Island_scaffold6_51738526_51739223 | NA | CGI | 5 |
| CpG_Island_scaffold6_532352_532890 | NA | CGI | 5 |
| CpG_Island_scaffold70_247298_247830 | NA | CGI | 5 |
| CpG_Island_scaffold70_260836_261624 | NA | CGI | 5 |
| CpG_Island_scaffold70_2881154_2882127 | NA | CGI | 5 |
| CpG_Island_scaffold70_7355052_7355565 | NA | CGI | 5 |
| CpG_Island_scaffold70_7680153_7681150 | NA | CGI | 5 |
| CpG_Island_scaffold70_7940696_7941195 | NA | CGI | 5 |
| CpG_Island_scaffold70_8554722_8555222 | NA | CGI | 5 |
| CpG_Island_scaffold71_3202664_3203233 | NA | CGI | 5 |
| CpG_Island_scaffold722_52466_53636 | NA | CGI | 5 |
| CpG_Island_scaffold72_4402441_4402968 | NA | CGI | 5 |
| CpG_Island_scaffold72_8243822_8244675 | NA | CGI | 5 |
| CpG_Island_scaffold72_8398493_8399296 | NA | CGI | 5 |
| CpG_Island_scaffold75_2767662_2768255 | NA | CGI | 5 |
| CpG_Island_scaffold767_52007_52855 | NA | CGI | 5 |
| CpG_Island_scaffold7_40753712_40754823 | NA | CGI | 5 |
| CpG_Island_scaffold7_44576871_44577379 | NA | CGI | 5 |
| CpG_Island_scaffold7_45137297_45137812 | NA | CGI | 5 |
| CpG_Island_scaffold7_46050215_46051315 | NA | CGI | 5 |
| CpG_Island_scaffold7_58050842_58051341 | NA | CGI | 5 |
| CpG_Island_scaffold7_6825836_6826492 | NA | CGI | 5 |
| CpG_Island_scaffold7_9639328_9640162 | NA | CGI | 5 |
| CpG_Island_scaffold80_4328540_4329859 | NA | CGI | 5 |
| CpG_Island_scaffold80_5495490_5496436 | NA | CGI | 5 |
| CpG_Island_scaffold81_3028856_3029355 | NA | CGI | 5 |
| CpG_Island_scaffold81_4000758_4001667 | NA | CGI | 5 |
| CpG_Island_scaffold81_7288929_7289608 | NA | CGI | 5 |
| CpG_Island_scaffold82_6540746_6542257 | NA | CGI | 5 |
| CpG_Island_scaffold83_2279885_2280860 | NA | CGI | 5 |
| CpG_Island_scaffold83_7365502_7366064 | NA | CGI | 5 |
| CpG_Island_scaffold86_6947382_6948128 | NA | CGI | 5 |
| CpG_Island_scaffold87_3467487_3467996 | NA | CGI | 5 |
| CpG_Island_scaffold89_6117772_6118771 | NA | CGI | 5 |
| CpG_Island_scaffold8_20032639_20033299 | NA | CGI | 5 |
| CpG_Island_scaffold8_22030348_22030873 | NA | CGI | 5 |
| CpG_Island_scaffold8_23857680_23858829 | NA | CGI | 5 |
| CpG_Island_scaffold8_5374668_5375255 | NA | CGI | 5 |
| CpG_Island_scaffold8_7260460_7260991 | NA | CGI | 5 |
| CpG_Island_scaffold93_1296190_1297319 | NA | CGI | 5 |
| CpG_Island_scaffold972_17910_18543 | NA | CGI | 5 |
| CpG_Island_scaffold972_20752_21286 | NA | CGI | 5 |
| CpG_Island_scaffold9_1094625_1095134 | NA | CGI | 5 |
| CpG_Island_scaffold9_6222873_6223737 | NA | CGI | 5 |
| CpG_Island_scaffold9_949519_950240 | NA | CGI | 5 |

CDS: Coding sequence; CGI: CpG island; tss: Transcription start side.

**Table S2.** Annotated DMRs in “experiment H” present in at least four of five father-sorted son groups (F1L_C_ vs F1L_H_) with at least one annotated gene of protein-coding genes present (applied in Figure 4).

| **ID** | **Gene Name** | **Annotation Type** | **No. of Father-Son Groups** |
| --- | --- | --- | --- |
| ENSCPOG00000023463_tss | *SNCG* | tss | 4 |
| ENSCPOG00000026310_promoter | unknown gene | promoter | 5 |
| ENSCPOG00000025943_promoter | *ARHGAP25* | promoter | 4 |
| ENSCPOG00000025820_promoter | *IL12RB1* | promoter | 4 |
| ENSCPOG00000025541_promoter | *GPR37L1* | promoter | 4 |
| ENSCPOG00000025428_promoter | *MAP2K2* | promoter | 4 |
| ENSCPOG00000025389_promoter | *BCL2L10* | promoter | 5 |
| ENSCPOG00000025334_promoter | *ICMT* | promoter | 4 |
| ENSCPOG00000024373_promoter | *TTC31* | promoter | 4 |
| ENSCPOG00000024356_promoter | *PPP1R18* | promoter | 5 |
| ENSCPOG00000023502_promoter | *Ccer1* | promoter | 5 |
| ENSCPOG00000023391_promoter | *COL5A1* | promoter | 4 |
| ENSCPOG00000022128_promoter | *MAL* | promoter | 4 |
| ENSCPOG00000021900_promoter | *Rac2* | promoter | 5 |
| ENSCPOG00000020508_promoter | *C5orf45* | promoter | 5 |
| ENSCPOG00000019570_promoter | *FZR1* | promoter | 4 |
| ENSCPOG00000013933_promoter | *SOGA2* | promoter | 4 |
| ENSCPOG00000012427_promoter | *PLD6* | promoter | 5 |
| ENSCPOG00000011847_promoter | *KCND2* | promoter | 4 |
| ENSCPOG00000009704_promoter | *ABLIM2* | promoter | 4 |
| ENSCPOG00000006486_promoter | *TICAM1* | promoter | 4 |
| ENSCPOG00000003083_promoter | unknown gene | promoter | 4 |
| ENSCPOG00000002302_promoter | *TMED1* | promoter | 4 |
| ENSCPOG00000001582_promoter | *U2AF2* | promoter | 5 |
| ENSCPOG00000023463_start_codon | *SNCG* | codon | 4 |
| ENSCPOG00000026460_CDS | *C2orf54* | CDS | 4 |
| ENSCPOG00000025080_CDS | *CHD5* | CDS | 4 |
| ENSCPOG00000024746_CDS | *GOT1L1* | CDS | 4 |
| ENSCPOG00000024356_CDS | *PPP1R18* | CDS | 5 |
| ENSCPOG00000024345_CDS | *SAPCD1* | CDS | 4 |
| ENSCPOG00000024187_CDS | *Padi6* | CDS | 4 |
| ENSCPOG00000023974_CDS | *ZCCHC3* | CDS | 4 |
| ENSCPOG00000023599_CDS | *HGFAC* | CDS | 4 |
| ENSCPOG00000023486_CDS | *CCDC142* | CDS | 4 |
| ENSCPOG00000023463_CDS | *SNCG* | CDS | 4 |
| ENSCPOG00000023234_CDS | *A1BG* | CDS | 4 |
| ENSCPOG00000022556_CDS | *RGR* | CDS | 4 |
| ENSCPOG00000022172_CDS | *ABCB9* | CDS | 4 |
| ENSCPOG00000021785_CDS | *C10orf105* | CDS | 5 |
| ENSCPOG00000021071_CDS | *C6orf25* | CDS | 4 |
| ENSCPOG00000020425_CDS | *SHROOM1* | CDS | 4 |
| ENSCPOG00000019906_CDS | *FAM117A* | CDS | 4 |
| ENSCPOG00000019861_CDS | *C9orf142* | CDS | 4 |
| ENSCPOG00000019765_CDS | *GAS2L1* | CDS | 5 |
| ENSCPOG00000019701_CDS | *ASIC3* | CDS | 5 |
| ENSCPOG00000015568_CDS | *LRRC24* | CDS | 4 |
| ENSCPOG00000015432_CDS | *KCNF1* | CDS | 5 |
| ENSCPOG00000015413_CDS | *C6orf136* | CDS | 4 |
| ENSCPOG00000015232_CDS | *LAMP3* | CDS | 4 |
| ENSCPOG00000015231_CDS | *KBTBD5* | CDS | 4 |
| ENSCPOG00000015128_CDS | *CSF3* | CDS | 5 |
| ENSCPOG00000014919_CDS | *SH3TC2* | CDS | 5 |
| ENSCPOG00000014435_CDS | *DNAJB1* | CDS | 5 |
| ENSCPOG00000014423_CDS | *ARFRP1* | CDS | 4 |
| ENSCPOG00000014036_CDS | *SPEN* | CDS | 4 |
| ENSCPOG00000013467_CDS | *Actb* | CDS | 5 |
| ENSCPOG00000012950_CDS | *PIK3CD* | CDS | 4 |
| ENSCPOG00000012910_CDS | *DENND4B* | CDS | 4 |
| ENSCPOG00000012781_CDS | *RBM38* | CDS | 4 |
| ENSCPOG00000012432_CDS | *DOCK4* | CDS | 5 |
| ENSCPOG00000012318_CDS | *GARNL3* | CDS | 4 |
| ENSCPOG00000011702_CDS | *ABCD1* | CDS | 4 |
| ENSCPOG00000011364_CDS | *INPP5D* | CDS | 4 |
| ENSCPOG00000011162_CDS | *TFR2* | CDS | 4 |
| ENSCPOG00000010952_CDS | *B4GALNT3* | CDS | 5 |
| ENSCPOG00000010016_CDS | *EXOC3L4* | CDS | 4 |
| ENSCPOG00000009813_CDS | *ASGR1* | CDS | 4 |
| ENSCPOG00000009742_CDS | *PCDHA5* | CDS | 4 |
| ENSCPOG00000009639_CDS | *RLBP1* | CDS | 4 |
| ENSCPOG00000009223_CDS | *GRB10* | CDS | 4 |
| ENSCPOG00000009196_CDS | *FAM131A* | CDS | 4 |
| ENSCPOG00000008349_CDS | *THEG* | CDS | 4 |
| ENSCPOG00000008083_CDS | *EFEMP2* | CDS | 5 |
| ENSCPOG00000007922_CDS | *C20orf85* | CDS | 4 |
| ENSCPOG00000006927_CDS | *Babam1* | CDS | 4 |
| ENSCPOG00000006097_CDS | *PROM2* | CDS | 4 |
| ENSCPOG00000005553_CDS | *IFI30* | CDS | 4 |
| ENSCPOG00000004968_CDS | *KBTBD10* | CDS | 4 |
| ENSCPOG00000004915_CDS | *SOX15* | CDS | 5 |
| ENSCPOG00000004736_CDS | *EDN3* | CDS | 4 |
| ENSCPOG00000004082_CDS | *DOLK* | CDS | 4 |
| ENSCPOG00000003966_CDS | *GPATCH2* | CDS | 4 |
| ENSCPOG00000003722_CDS | *SRSF3* | CDS | 4 |
| ENSCPOG00000003539_CDS | *CELF3* | CDS | 4 |
| ENSCPOG00000003371_CDS | *STAB2* | CDS | 4 |
| ENSCPOG00000003362_CDS | *ETV4* | CDS | 4 |
| ENSCPOG00000003239_CDS | *APBA1* | CDS | 4 |
| ENSCPOG00000003125_CDS | *BTG4* | CDS | 4 |
| ENSCPOG00000002716_CDS | *CYP21A2* | CDS | 4 |
| ENSCPOG00000002024_CDS | *CMBL* | CDS | 4 |
| ENSCPOG00000001921_CDS | *TOPAZ1* | CDS | 4 |
| ENSCPOG00000001618_CDS | *KCNH6* | CDS | 5 |
| ENSCPOG00000001215_CDS | *FGD2* | CDS | 4 |
| ENSCPOG00000001014_CDS | *GSTO1* | CDS | 4 |
| ENSCPOG00000000970_CDS | *MAP2K5* | CDS | 4 |
| ENSCPOG00000000944_CDS | *SEMA4G* | CDS | 5 |
| ENSCPOG00000000682_CDS | *PTHR11347_SF105* | CDS | 4 |
| ENSCPOG00000000131_CDS | *CCDC106* | CDS | 5 |
| CpG_Island_scaffold96_429073_429726 | NA | CGI | 5 |
| CpG_Island_scaffold91_1274441_1274969 | NA | CGI | 4 |
| CpG_Island_scaffold9_11449859_11450361 | NA | CGI | 4 |
| CpG_Island_scaffold72_8633449_8633948 | NA | CGI | 4 |
| CpG_Island_scaffold72_7952424_7953019 | NA | CGI | 4 |
| CpG_Island_scaffold72_7756727_7757274 | NA | CGI | 4 |
| CpG_Island_scaffold71_2860811_2861457 | NA | CGI | 4 |
| CpG_Island_scaffold70_8735848_8736348 | NA | CGI | 5 |
| CpG_Island_scaffold70_7104123_7104758 | NA | CGI | 4 |
| CpG_Island_scaffold70_31508_32009 | NA | CGI | 4 |
| CpG_Island_scaffold7_58311390_58311892 | NA | CGI | 4 |
| CpG_Island_scaffold7_57665075_57665574 | NA | CGI | 5 |
| CpG_Island_scaffold7_49534679_49535273 | NA | CGI | 4 |
| CpG_Island_scaffold7_47571060_47572660 | NA | CGI | 5 |
| CpG_Island_scaffold7_46308352_46309087 | NA | CGI | 4 |
| CpG_Island_scaffold65_983959_985023 | NA | CGI | 5 |
| CpG_Island_scaffold65_4665560_4666457 | NA | CGI | 5 |
| CpG_Island_scaffold62_4399648_4400359 | NA | CGI | 4 |
| CpG_Island_scaffold61_9058152_9059057 | NA | CGI | 4 |
| CpG_Island_scaffold61_9054899_9055727 | NA | CGI | 5 |
| CpG_Island_scaffold6_3158393_3159734 | NA | CGI | 4 |
| CpG_Island_scaffold6_24424657_24425156 | NA | CGI | 4 |
| CpG_Island_scaffold53_11981249_11981757 | NA | CGI | 4 |
| CpG_Island_scaffold53_10797058_10798420 | NA | CGI | 5 |
| CpG_Island_scaffold52_9916819_9917394 | NA | CGI | 4 |
| CpG_Island_scaffold52_8371925_8372880 | NA | CGI | 4 |
| CpG_Island_scaffold509_11360_11859 | NA | CGI | 4 |
| CpG_Island_scaffold429_95158_95955 | NA | CGI | 4 |
| CpG_Island_scaffold42_13324813_13325557 | NA | CGI | 4 |
| CpG_Island_scaffold42_13058586_13059146 | NA | CGI | 4 |
| CpG_Island_scaffold42_13031930_13032510 | NA | CGI | 5 |
| CpG_Island_scaffold406_244786_245548 | NA | CGI | 4 |
| CpG_Island_scaffold406_208986_210218 | NA | CGI | 5 |
| CpG_Island_scaffold4_5260524_5261114 | NA | CGI | 4 |
| CpG_Island_scaffold4_33132022_33132631 | NA | CGI | 4 |
| CpG_Island_scaffold368_14997_15994 | NA | CGI | 4 |
| CpG_Island_scaffold334_141453_142390 | NA | CGI | 5 |
| CpG_Island_scaffold33_18212468_18213158 | NA | CGI | 4 |
| CpG_Island_scaffold32_22951600_22952445 | NA | CGI | 5 |
| CpG_Island_scaffold314_124417_125025 | NA | CGI | 4 |
| CpG_Island_scaffold314_110299_111344 | NA | CGI | 4 |
| CpG_Island_scaffold312_476475_476989 | NA | CGI | 4 |
| CpG_Island_scaffold31_9537238_9537980 | NA | CGI | 5 |
| CpG_Island_scaffold31_22264471_22265675 | NA | CGI | 5 |
| CpG_Island_scaffold304_315855_316406 | NA | CGI | 4 |
| CpG_Island_scaffold304_311773_312492 | NA | CGI | 4 |
| CpG_Island_scaffold30_8415571_8416703 | NA | CGI | 4 |
| CpG_Island_scaffold30_2140105_2140644 | NA | CGI | 5 |
| CpG_Island_scaffold3_19883157_19884591 | NA | CGI | 4 |
| CpG_Island_scaffold27_2619059_2619665 | NA | CGI | 4 |
| CpG_Island_scaffold27_1480595_1481094 | NA | CGI | 4 |
| CpG_Island_scaffold25_27701476_27702098 | NA | CGI | 4 |
| CpG_Island_scaffold25_25802901_25803453 | NA | CGI | 5 |
| CpG_Island_scaffold25_17835999_17836504 | NA | CGI | 4 |
| CpG_Island_scaffold25_13488570_13489078 | NA | CGI | 5 |
| CpG_Island_scaffold24_9405984_9406483 | NA | CGI | 5 |
| CpG_Island_scaffold24_2941038_2941806 | NA | CGI | 4 |
| CpG_Island_scaffold23_29662325_29662882 | NA | CGI | 5 |
| CpG_Island_scaffold175_119431_119973 | NA | CGI | 5 |
| CpG_Island_scaffold168_1884123_1884833 | NA | CGI | 4 |
| CpG_Island_scaffold160_2020178_2020757 | NA | CGI | 4 |
| CpG_Island_scaffold16_36817085_36817599 | NA | CGI | 4 |
| CpG_Island_scaffold1592_2905_3423 | NA | CGI | 4 |
| CpG_Island_scaffold15_41196963_41197498 | NA | CGI | 4 |
| CpG_Island_scaffold15_37852249_37852748 | NA | CGI | 4 |
| CpG_Island_scaffold15_34294514_34295207 | NA | CGI | 4 |
| CpG_Island_scaffold15_34244619_34245449 | NA | CGI | 4 |
| CpG_Island_scaffold15_32260044_32260722 | NA | CGI | 4 |
| CpG_Island_scaffold13_4596760_4597446 | NA | CGI | 4 |
| CpG_Island_scaffold13_45330723_45331619 | NA | CGI | 5 |
| CpG_Island_scaffold125_3084321_3085619 | NA | CGI | 5 |
| CpG_Island_scaffold12_22302260_22302759 | NA | CGI | 4 |
| CpG_Island_scaffold12_21225529_21226030 | NA | CGI | 4 |
| CpG_Island_scaffold117_1704075_1705077 | NA | CGI | 5 |
| CpG_Island_scaffold1156_8353_8964 | NA | CGI | 5 |
| CpG_Island_scaffold115_4041100_4041661 | NA | CGI | 4 |
| CpG_Island_scaffold114_983315_984202 | NA | CGI | 4 |
| CpG_Island_scaffold11_41524895_41526146 | NA | CGI | 4 |
| CpG_Island_scaffold104_2861304_2861812 | NA | CGI | 4 |
| CpG_Island_scaffold102_2262290_2262791 | NA | CGI | 4 |
| CpG_Island_scaffold10_34751859_34752389 | NA | CGI | 4 |
| CpG_Island_scaffold10_34119306_34119891 | NA | CGI | 5 |
| CpG_Island_scaffold0_81473993_81474499 | NA | CGI | 4 |

CDS: Coding sequence; CGI: CpG island; tss: Transcription start side.
